# From Anti-EBV Immune Responses to the EBV Diseasome via Cross-reactivity

**DOI:** 10.1055/s-0040-1715641

**Published:** 2020-08-31

**Authors:** Darja Kanduc, Yehuda Shoenfeld

**Affiliations:** 1 Department of Biosciences, Biotechnologies, and Biopharmaceutics, University of Bari, Bari, Italy; 2 Zabludowicz Center for Autoimmune Diseases, Sheba Medical Center, Tel-Aviv University School of Medicine, Tel-Hashomer, Israel; 3 I.M. Sechenov First Moscow State Medical University of the Ministry of Health of the Russian Federation, Sechenov University, Moscow, Russia

**Keywords:** EBV epitopes, systemic lupus erythematosus, cross-reactivity, autoimmunity, negative selection, self-reactive lymphocytes, pathogenic autoantibodies

## Abstract

Sequence analyses highlight a massive peptide sharing between immunoreactive Epstein-Barr virus (EBV) epitopes and human proteins that—when mutated, deficient or improperly functioning—associate with tumorigenesis, diabetes, lupus, multiple sclerosis, rheumatoid arthritis, and immunodeficiencies, among others. Peptide commonality appears to be the molecular platform capable of linking EBV infection to the vast EBV-associated diseasome via cross-reactivity and questions the hypothesis of the “negative selection” of self-reactive lymphocytes. Of utmost importance, this study warns that using entire antigens in anti-EBV immunotherapies can associate with autoimmune manifestations and further supports the concept of peptide uniqueness for designing safe and effective anti-EBV immunotherapies.

## Introduction

The connection between Epstein-Barr virus (EBV) and Burkitt's lymphoma (BL) was discovered in 1964. 1 Almost contemporaneously, high anti-EBV antibody levels were found in BL. 2 3 Since then, EBV infection has been associated with a wide spectrum of malignancies that, besides BL, comprehends different types of lymphomas, nasopharyngeal carcinoma (NPC), breast and brain cancer, and oral hairy leukoplakia, 4 5 6 7 8 among others. In addition, EBV has been implicated in a wide variety of diseases, including systemic lupus erythematosus (SLE), Sjögren's syndrome, multiple sclerosis (MS), myasthenia gravis (MG), rheumatoid arthritis (RA), autoimmune thyroid disorders, inflammatory bowel disease, celiac diseases, diabetes, Parkinson's disease, myopericarditis, dilated cardiomyopathy, and even death. 9 10 11 12 13 14

At the same time, anti-EBV antibody level was found to be higher in BL patients than in control subjects. 3 15 16 17 High level of anti-EBV immunoglobulin G antibodies were also found in subjects with NPC, 18 19 20 21 with IgG reactivity increasing significantly with tumor stage 21 ; Hodgkin and non-Hodgkin lymphomas 22 23 24 ; precancerous gastric lesions 25 ; MS 26 27 28 29 ; RA 30 31 32 ; MG 33 34 ; and SLE, 29 31 34 among others. In general, high antibody titers to EBV appeared to be related to a worse prognosis, a phenomenon that has been described by Coutinho's laboratory 35 as “the advantage of being low-respondents.” Currently, measurement of increased anti-EBV antibody titers is utilized to predict, to detect, and to monitor the progression of EBV-related cancers and progression of the various EBV-induced diseases. 36 37 38

Today, in front of such well known clinical context, the molecular mechanism(s) by which anti-EBV immune responses relate to the EBV diseasome, from lymphomas to Parkinson's disease, are still obscure. From a logical point of view, a central question remains unanswered and perhaps, as far as we know, has never been clearly posed: why the powerful anti-EBV immune responses herald cancers, autoimmune diseases, and death instead of eradicating the viral infection and re-establishing a healthy status?

In the clinical frame exposed above and on the basis of previous scientific reports 39 40 41 42 that have detailed a high level of peptide sharing between EBV and human proteins involved in crucial functions, this study investigates whether the immune responses that accompany active EBV infection have the potential to cross-react with and damage human proteins that, when altered, can lead to various cancer and autoimmune diseases. That is, the thesis is explored according to which the anti-EBV immune responses that should be a “protective defense” from EBV infection actually cross-react with human proteins, in this way setting up an anti-human protein assault with catastrophic pathologic sequelae in the body. Specifically, the present study used the pentapeptide as an antigenic and immunogenic unit, 43 44 45 46 47 48 and analyzed 3,197 experimentally validated immunoreactive EBV-derived epitopes for pentapeptide matches with the human proteome. Data are reported on a vast peptide sharing between EBV epitopes and proteins involved in tumorigenesis, autoimmune disorders, diabetes, and death, among others. The data suggest that cross-reactivity is the mechanism underlying the causal connection between EBV infection, immune response, and the EBV-associated diseases.

## Methods

An EBV immunome formed by 3,197 immunopositive linear epitopes was assembled from Immune Epitope DataBase (IEDB, www.iedb.org ). 49 The immunopositive EBV epitopes are listed in Supplementary Table S1 (available in the online version). EBV epitope sequences were dissected into pentapeptides overlapped each other by four amino acid (aa) residues. The resulting 11,564 pentapeptides were analyzed for occurrence(s) within the human proteome using Pir Peptide Match Program. 50 Proteins related to EBV-induced diseases were annotated. UniProtKB database ( http://www.uniprot.org/ ) 51 PubMed, and OMIM resources were used.

**Table 1:** Numerical description of the pentapeptide sharing between the set of 3,197 immunopositive EBV epitopes and the human proteome

Pentapeptides composing the 3,197 EBV epitope immunome	11,564
EBV epitope pentapeptides not shared with the human proteome	798
EBV epitope pentapeptides shared with the human proteome	10,766
Human proteins sharing pentapeptides with EBV epitopes	18,744
Occurrences of EBV epitope pentapeptides in the human proteome (including multiple occurrences)	137,805

Abbreviation: EBV, Epstein-Barr virus.

## Results

### Quantitation of the Peptide Sharing between EBV Epitopes and the Human Proteome

Following matching analyses of the 11,564 pentapeptides composing the 3,197 experimentally validated immunoreactive EBV epitopes, it was found that almost all of the epitope-derived pentapeptides (i.e., 93%) are widespread among thousands of human proteins ( Table 1 ). From a mathematical point of view, if one considers that the probability of a pentapeptide to occur in two proteins is 20 ^−5^ (or 1 out of 3,200,000 or 0.0000003125), then the peptide overlap existing between the EBV immunome and the human proteome is staggering.

### Distribution of the Peptide Sharing among EBV Epitopes

A synthetic snapshot (i.e., 201 EBV epitopes) of the immunoreactive peptide sharing is shown in Table 2 , where peptide sequences shared with the human proteins are given in capital format and peptide fragments uniquely present in EBV are given with aa in small bold format. Table 2 clearly shows that the immunoreactive EBV epitopes are predominantly composed by peptide sequences common to human proteins.

**Table 2:** Pentapeptide sharing between 201 immunoreactive EBV epitopes and human proteins a

IEDB ID b	EPITOPE c d	IEDB ID b	EPITOPE c d	IEDB ID b	EPITOPE c d
950	AEGLRALLARSHVER	45499	NPTQAPVIQLVHAVY	127195	TEMYIMYAM
1518	AGGAGAGGGAGGA	46498	NVTQVGSEPISPEIG	127369	WEMRAGREI
1716	AGVFVYGGSKTSLYN	47613	PGAPGGSGSGP	127392	WPTPKTHPV
2390	ALA **ipqcr** L	47760	PGTGPGNGLGEKGDT	127408	**yamai** RQAI
2742	ALLVLYSFAL	48320	PLFDRKSDAK	137773	YNLRRGIAL
2743	ALLVLYSFALMLIIIILIIF	48486	PLSRLPFGM	138854	GAGAGAGA
3005	ALWNLHGQALFLGIVL	48852	PPPGRRP **ffhpv** GE	138856	GRGRGRGR
3600	**apify** PPVL	48876	PPP **qapyq** GY	138882	MTAASYARY
3782	APRLPDDPI	49864	PVFDRKSDAK	138873	LMARRARSL
3951	AQEILSDNSEISVFPK	50298	QAKWRLQTL	141342	LLDFVRMGV
5316	AVFDRKSDAK	51685	QNGALAINTF	144799	TLNLT
5317	AVFDRKSVAK	51946	QPRAPIRPI	167590	GPQRR
5326	AVFNRKSDAK	52142	QQ **rpvmfv** SRVPAKK	186702	PQPRAPIRPIPT
5439	AVLL **heesm**	53195	RARGRGRGRGEKRP	191290	FIVFLQTHI
8120	DEPASTEPVHDQLL	54367	RKIYDLIEL	227777	HPVAEADYFEY
8905	DKI **vqapify** PPVLQ	54728	RLRAEAQVK	230640	ASDYSQGAF
9644	DP **hgpvq** LSYYD	55251	R **ppifi** RLL	230798	FYPPVLQPI
10448	DTPLIPLTIF	55298	RPQKRPSCI	231136	LAYA **rgqam**
10858	DYDASTESEL	55327	RPRPPARSL	231402	RRVRRRVLV
10963	DYSQGAFTPL	55529	RRARSLSAERY	231547	TVFY **nippm**
11804	EENLLDFVRF	55619	RRIYDIEL	231696	YRTATLRTL
12183	EGGVGWRHW	56506	RYAREAEVRF	231699	YSQGAFTPL
13628	EPDVPPGAIEQGPAD	56523	RYEDPDAPL	231800	AQPAPQAPY
16876	FLRGRAYGI	56650	RYSIFFDY	231839	DSIMLTATF
17110	FMVFLQTHI	56651	RYSIFFDYM	231840	DTR **aidqf** F
17600	FRKAQIQGL	57755	SFFDRKSDAK	231880	FLQRTDLSY
18328	FVYGGSKTSL	59084	SLFDRKSDAK	231966	HVIQNAFRK
18438	FY **nippm** PL	59432	SLREWLLRI	232020	KPWLRAHPV
18946	GDDGDDGDEGGDGDE	62305	SVRDRLARL	232021	KQRKPGGPW
19674	GGAGGAGGAGAGGGAG	67456	TYSAGIVQI	232030	KTIG **nfkpy**
19737	GGGAGAGGAGAGGGGR	68561	VFSDGRVAC	232074	LPTPMQLAL
20023	GGSKTSLYNLRRGTA	69558	VLKDAIKDL	232076	LQALSNLIL
21719	GPPAA	70251	VPAPAGPIV	232078	LQSSSYPGY
21723	GPPAAGPPAAGPPAA	70624	VQPPQLTQV	232080	LS **aeryt** LF
21870	GQGGSPTAM	70932	VSFI **efvgw**	232081	LSVIPSNPY
22159	GRPAVFDRKSDAKST	71968	VYAASFSPNL	232086	LTQAAGQAF
22976	GVFVYGGSKTSLYNL	72028	VYGGSKTSL	232095	LVSSGNTLY
23324	G **ydvgh** GPL	72251	WAPSV	232096	LVSSSAPSW
23449	GYRTATLRTL	73221	WVPSV	232103	MEQRVMATL
23994	H **hiwqnll**	74120	**yhliv** DTDSL	232177	QEPGPVGPL
24170	HLAAQGMAY	75188	YNLRRGTAL	232178	QEQLSDTPL
24533	HPV **geady**	75189	YNLRRGTALAIPQ	232199	RESIVCYFM
24666	HRCQAIRK	75356	YPL **heqhg** M	232214	RLHRLLLMR
24667	HRCQAIRKK	75360	YPLHKQHGM	232232	RPAP **pkiam**
26480	IIFIFRRDLLCPLGAL	75731	YSFALMLIIIILIIFIFRRD	232276	SEPCEALDL
26538	IIIILIIFI	79634	QPRAPIRPIT	232308	SQISNTEMY
27103	ILIIFIFRRDLLCPLGALCI	93251	LLARSHVER	232332	TE **dnvppwl**
27301	ILRQLLTGGVKKGRP	94034	THIFAEVLKD	232416	VTFSAGTFK
27375	ILTDFSVIK	97317	**fwemr** AGREITQ	232419	VTTQRQSVY
29618	IYLLEMLWRL	98084	GVFVYGGSK	232427	**waqig** HIPY
30430	KEHVIQNAF	101654	FVYGGSKTSLY	232437	WQRRYRRIY
30431	KEHVIQNAFRK	101878	LQTHIFAEV	232473	YQEPPAHGL
33207	KR **ppifi** RR	102253	YPL **heqyg** M	237896	QTAAAVVF
33866	KTSLYNLRRGTALA	106084	RPRSPSSQSSSSGSPPRRP	237920	RYKNRVASR
35162	LDFVRFMGV	107724	AARQRLQDI	540571	QPRLTPPQPL
35533	LEKARGSTY	107869	GPKVKRPPI	540583	RPTELQPTP
37153	LLDFVRFMGV	108006	LLDFV **rfmgy**	540628	TSSPSMPEL
38514	LPGPQVTAVLLHEES	108191	VMATLLPPV	548981	LLDFVRFMG
39102	**lrgkw** QRRYR	118970	PPPGRRP	548987	NGALAINTF
39634	LSRLPFGMA	124861	WNLHGQALFL	548994	QNGALAINT
41113	MARRARSLS **aeryt** L	126528	LA **samrm** LW	595247	FGLVLFPAQI
41147	MATLLPPVPQQPRAG	126967	RPRP **rtpew**	653929	AAQGMAY
41841	**mkkaw** LSRAQQADAG	126980	RRAALSGHL	672845	PIFIRRL
42525	MSDEGPGTGPGNGLG	126985	RRLHRLLLM	674203	R **amsfi** ATY
42941	MVFLQTHIFAEVLKD	126986	RRRRRRAAL	675184	R **ppifi** R
44181	NIAEGLRAL	126991	RRYRRIYDL	676208	RRIYDLI
45378	N **pkfen** IAEGLRALL	127118	SQAAFGLPI	695961	QAPYPGYEE

Abbreviations: EBV, Epstein-Barr virus; IEDB, Immune Epitope DataBase.

a Epitopes assembled from IEDB ( www.iedb.org ). 49 Epitope experimental details and references are available at www.iedb.org .

b Epitopes listed according to IEDB ID number. 49

c Epitope sequences given in 1-letter code.

d Pentapeptides shared between EBV epitopes and human proteins are given in capital letters, while pentapeptides present only in EBV are given in bold small format.

Immunologically, Tables 1 and 2 document that the experimentally validated immunoreactive EBV epitopes mostly consist of pentapeptides that also occur in human proteins, in this way indicating a highest cross-reactivity potential, given the fact that a pentapeptide is a minimal immune determinant that contains the immunological information in terms of both immunogenicity and antigenicity. 43 44 45 46 47 48

### The Pathological Implications of the Peptide Sharing between EBV Epitopes and Human Proteins: Lymphomas and Leukemias

Numerous cancer-related proteins share peptides with the here analyzed self-reactive EBV epitopes. Reasons of space do not permit a detailed peptide-by-peptide description of the sharing and only a few examples are described in Tables 3 and 4 . Specifically, Table 3 shows the peptide sharing between the immunoreactive EBV epitopes and human proteins that—when mutated, modified, improperly functioning or deficient—are implicated in lymphomagenesis/leukemogenesis. 52 53 54 55 56 57 58 59 60 61 62 63 64 65 66 67 68 69 70 71 72 73 74 75 76 77 78 79 80 81 82 83 84 85 86 87 88 89 90 91 It can be seen that the extent of the peptide sharing is very high and comes to the fore with glaring evidence when focusing on histone-lysine *N* -methyltransferase 2D (KMT2D), the disruption of which perturbs germinal center B cell development and promotes lymphomagenesis. 77 78 KMT2D alterations are involved in follicular lymphoma and diffuse large B-cell lymphoma, 92 cutaneous T-cell lymphoma and Sézary syndrome, 93 ocular adnexal MALT-type marginal zone lymphomas, 79 and chronic myeloid leukemia. 94 Moreover, KMT2D alterations are involved in intraocular medulloepithelioma, 95 small cell lung cancer, 96 bladder cancer, 97 98 and non-small-cell lung cancer. 99 Of not less importance, alterations of KMT2D have a causal role in Kabuki syndrome 100 that is characterized by skeletal and visceral abnormalities and cardiac anomalies, 101 hyperinsulinism, 102 epilepsy, 103 desmoid fibromatosis, 104 immunopathological manifestations, 105 lupus, 106 and oriental alterations, 107 among others.

**Table 3:** Pentapeptide sharing between immunoreactive EBV epitopes and human proteins implicated in lymphomagenesis/leukemogenesis

Shared peptides	Lymphoma/Leukemia-related proteins containing EBV epitope peptide(s) a	Ref b
LSPLL, RRQKR, LALRA, KEVLE, LGLGD, GNLVT, SLESV, LPTLL, PETVP, ALYLQ, ARVKE, PSLKL, KILLA, NPETL, EGLKD, LYLQQ, QKRPS, VAKVA, DRHSD, LQAIG, LSQVC, RPSCI	ATM: Serine-protein kinase ATM	52
PLPPP, PPLPP, LPTLL, REAIL, AERHG, CKKDH	BANK1: B-cell scaffold protein with ankyrin repeats	53 54
EEEEE, PPLPP, GAGGG, AGAGG, GAGGA, AGGGA, GRGGG, PPPVS, LSAAS, PPLGP, PPVSP, EPGPA, PVSPG, SSLTP, TPPPQ, GDDDD, AVAQS, DPSLG, GNSST, PGLFP, SEPVE, DDAGG, DDDAG	BC11B: B-cell lymphoma/leukemia 11B	55 56 57
SSSEE, GPPSP, APAST, GPEAR, PCPQA, PQARL, RFIQA	BCL6B: B-cell/lymphoma 6 member B protein	58 59 60 61 62
SPSPP, PLPPV, GSGAG, PAGSL, PVPPP, EPGPA, EQASL, EGTRL, LDLDF, LNQNL, VLQKL	BIN1: Myc box-dependent-interacting protein 1	63
LLLLL, LRLLL, RLRLL, KEDDG, EGGQN	CADM1: Cell adhesion molecule 1	64
PPPPP, LPPPP, GSGSG, GGGSG, GAGGG, GSGGS, SGGSG, GGSGS, SGSGG, LPPVP, PPVPA, PVPPT, QQGSG, CTPGD, PYILD	CBL: E3 ubiquitin-protein ligase CBL	65
PPPLP, PPGPS, LSPLS, SSPQP, ATSGA, ENLLD, EENLL, SPVLG, AFEEV, GPQDP, YDAPG	CRTC2: CREB-regulated transcription coactivator 2	66
LLARL, GASGS, VAGLL, PLHAL, LARLR, SGASP, CGLLR, VPKPR, FIRRL, TDGKT, TPLLT, ALIKT, SSCNS	DAPK1: Death-associated protein kinase 1	67 68
EEEAE, FGLSR, SDLSR, SLESV, KAIEE, VIQLV, IIAVV, VMDLL, IAVVA, IKAIE, ESFTQ, QDVGA, RLFAA, TTGGK, VIKAI, SFTQG	EPHA7: Ephrin type-A receptor 7 (998 aa)	69
LLLLL, PPPPP, LPPPP, ALLLL, LALLL, PPPPS, PPLPP, FPPPP, SLSST, GSPPR, PPQVP, SPSDS, TLSPS, TSEPV, SEDDP, ESVDV, GTPPQ, TDGGG, TSVVQ, VYAAS, EDDPQ, PSELD, DLRPL, FVGDY, KGTPP, PRLFA, VCSVA, HSPVV, ILQIS, LYEAS, PYEAF	FAT1: Protocadherin Fat 1	70 71
PPPPP, GGGGG, EEEEA, AAAAV, SSSEE, GGSGS, GGGGD, RGGSG, GAPGG, ASGPG, LPGVP, VSPAV, PGGLG, VEAHV, GGDGD, LRAAT, ERPLA, FPEGV, GGDKV	HIC1: Hypermethylated in cancer 1 protein	72
PPRPP, RRRKG, ATAAA, SVSQP, AEVLK, LLQTE, SHTAT	KDM6A: Lysine-specific demethylase 6A	73 74
PPPPP, QPPPP, SPPPP, SSSSA, SSSAP, PTPPP, GAPAA, KKRKR, RGGRG, GGRGR, PPPPY, SSSAG, GRGGR, LPPTP, APPTP, PPLGP, PTPLP, SGSPP, PQPPL, SQASA, DDEDL, STSVP, LPGVS, SSGTA, LTPRP, RPRGA, RQRSR, SGLGT, TPRPP, TPRPS, TSVPS, VTLPL, DLILQ, GTPRP, TPRPV, IAVSS, LDTED, TPRPR, LGATI, SAPRK, EGVEV, LSPAN, LSSCP, MQPPP, SLIQL, AKIEA, EDLFG, EEVEN, QGVQV, TPRSQ, VEDLF, LGLYA, PQSGP, DSREG, VSTAD, GPADD, PADDP, QSLIQ, VFPKD, DTDSL, GTFKP, IPQTL, PLQHW, TGQGK, EQHGM, IDDNS, LRPQW, QRHSD, TFKPP, GPRHT	KMT2C: Histone-lysine *N* -methyltransferase 2C	75 76
EEEEE, QPPPP, PLPPP, SAAAA, LRLLL, PAPAA, PAAAP, PTPPP, APPAP, GRGRG, PPSPG, PSPGS, PSPPP, RGRGR, SPLLP, AAPPA, GPAGP, LLAAL, PAQPP, SLGLA, LAPSP, LSPLL, PGPAG, SPSQS, SQSSS, GGRGR, GLPPP, PQGPP, RLRLL, LPPTP, LRSLG, PTLLL, SPSSQ, TPPPS, ALAPS, EGLRA, GPQPP, PEPPT, PLTEP, SSGSP, AASED, APVAP, AVGPP, DDEEL, ESPAR, GAHGG, GPPRL, KKRKR, LTPRP, PALDD, PPPGR, PPQGP, PPQVP, PPTQH, PTLGK, SDEAE, SPLLG, TPHTK, APYPG, ARPPE, ASDRL, CPSLD, DAAAR, EERPP, EGEGD, EGPST, EPRLA, FPDTK, FPEGL, GPLAI, GPWDP, GTQDP, IKVIE, LGLYA, LRLTP, LSPVI, PLLTV, PMSPP, PPTHP, PPVPQ, PQPLM, PQQPM, PSRPQ, QALAP, QEPPP, QTNQA, RGAFG, RPEFV, SDALG, SPVTP, SQTEL, SRVPA, SYTDP, TGSGG, TTPAG	KMT2D: Histone-lysine *N* -methyltransferase 2D	77 78 79
GGGGG, GGGGS, GGGGA, AGGGG, GGSGG, GGGSG, GAGGG, GGAGG, AGAGA, PPPEP, LRALL, LALRA, LTPPS, RALLA, RLLLK, PQAPE, TPLDL, GPETR, RVGAD	NFKB2: Nuclear factor NF-kappa-B p100 subunit	80 81 82 83
AAAPA, GAAAS, PAPGL, LLGGG, TPSPS, SLPHP, PHLPP, GSPTA, PLTSE, RDSYA, TTLAA, YPGYA, HRDSY, SYPGY	PRDM1: PR domain zinc finger protein 1	84
EEEEE, PSPPP, APAAA, SPSPP, PSPSP, SPSPS, PLDLS, DEGEE, LDLSV, LLTPV, PTVSP, KQLLQ, VLDLS, LTPVT, TVSPS, VTEDL, AIEEE, TSEET, PAPTV, TPVTV, EAVSF, FKPPP, SFKPP, NIPQT, YSLRL, PALRD, RSQVK, PFVGD	PRDM2: domain zinc finger PR protein 2	85 86 87 88
SSSSA, SPLLP, SSSAP, LSPLL, GTPSG, LQSET, PVSRF, AEGKL, PLRPT	SOCS6: Suppressor of cytokine signaling 6	68
KKRKR, AGAAR, LQSLA, TSPTS, RSLLT, LSLVF, AGPSV, DPVHG, GPSVA, QATLG, TQLTQ, DLQDP, LEKQS, PVQGE, QERDV, PKTAS, PLTQP, NIEEF, TPHQP, SHETP	TET1: Methylcytosine dioxygenase TET1	89
PPPLP, PPPPS, SPPPP, SSSEE, ELLEK, SASGS, QSSHL, APGGS, LQAPG, KLSSL, PPSQL, APPSQ, HLLQH, QQASV, VTKQE, VTVLT, PPTQH, PVTVL, GIKRT	TET2: Methylcytosine dioxygenase TET2	90
PPPPP, LPPPP, PLPPP, PPPLP, PPLPP, PPPPS, GGGRG, APGGG, GLPAP, QPPPQ, PAPGP, PRGPP, PPSSG, SLGLA, LPAPG, LPPVP, PLPPV, PPPSR, GGRPG, PPPGR, DLRSL, VGPLS, PMPPP, SEGLV, SGNGP, ADIGA, DIGAP, GGDQG, PVGPL	WASP: Wiskott-Aldrich syndrome protein	91

a Human proteins reported by UniProt entry names.

b Further references on the function/disease association at www.uniprot.org , OMIM, and PubMed resources.

**Table 4:** Quantitative pentapeptide matching between immunoreactive EBV epitopes and human proteins related to various cancers and diseases

Pentapeptides:		Human proteins sharing pentapeptides with EBV epitopes, and disease involvement c d	Refs.
A a	B b		
3	−	ACHA: Acetylcholine receptor subunit α. MG.	117
7	−	ACHD: Acetylcholine receptor subunit delta. MG.	117
8	−	ACHE: Acetylcholine receptor subunit epsilon. MG.	117
9	11	ACHG: Acetylcholine receptor subunit gamma. MG.	117
31	42	AGRB1: Adhesion G protein-coupled receptor B1. Inhibits glioma growth.	118 119
15	−	AKA12: A-kinase anchor protein 12. MG autoantigen. Involved in breast cancer.	120
27	−	APC: Adenomatous polyposis coli protein. Relates to colorectal adenomas and breast cancer.	121 122 123
64	68	APCL: Adenomatous polyposis coli protein 2. Its repression promotes ovarian cancer.	123 124
57	68	ARI1A: AT-rich interactive domain-containing protein 1A. Bladder, colorectal, endometrial, esophageal, gastric, kidney, liver, lung, ovarian cancers.	108
68	92	ARI1B: AT-rich interactive domain-containing protein 1B. Liver cancer.	108
33	−	ARID2: AT-rich interactive domain-containing protein 2. Liver, lung, melanoma cancers.	108
23	−	BCOR: BCL-6 corepressor. Tumor suppressor in endometrial cancer and medulloblastoma.	108 125
9	−	C1S: Complement C1s subcomponent precursor. SLE.	126
20	−	CHD4: Chromodomain-helicase-DNA-binding protein 4. Endometrial cancer.	108
32	34	CHD6. Chromodomain-helicase-DNA-binding protein 6. Bladder cancer.	108
38	−	CHD8: Chromodomain-helicase-DNA-binding protein 8. glioblastoma.	108
10	−	CLAT: Choline O-acetyltransferase. Myasthenic syndrome.	127
17	−	CO4A: Complement C4-A precursor. SLE.	128
29	56	CO4A1: Collagen α-1(IV) chain. Tumor suppressor; anti-angiogenic.	129
17	−	CO4B: Complement C4-B precursor. SLE.	128
12	13	CUL7: Cullin-7. 3M syndrome with growth restriction, skeletal abnormalities and dysmorphisms.	130
25	26	DCC: Netrin receptor DCC. Required for axon guidance. Colorectal cancer suppressor.	131
16	66	DMBT1: Deleted in malignant brain tumors 1 protein. Suppressed in human lung cancer.	132 133
59	−	DYST: Dystonin. Bullous pemphigoid.	134 135
42	−	FAT4: Protocadherin Fat 4. Involved in hepatocellular carcinoma. and in gastric cancer risk.	136 137
34	38	FUBP2: Far upstream element-binding protein 2.	138
11	−	IGF1R: Insulin-like growth factor 1 receptor. Intrauterine and postnatal growth retardation.	139
14	−	INSR: Insulin receptor. Insulin resistance syndrome with pineal hyperplasia.	140
13	−	INSR2: Insulin, isoform 2. Diabetes.	141
27	31	IRS1: Insulin receptor substrate 1. Diabetes. cognitive impairment and Alzheimer's disease.	142
42	45	IRS2: Insulin receptor substrate 2. Diabetes. cognitive impairment and Alzheimer's disease.	142 143 144
38	50	IRS4: Insulin receptor substrate 4. Diabetes. cognitive impairment and Alzheimer's disease.	142
20	−	KDM5A: Lysine-specific demethylase 5A. Intellectual disability. Inhibits glioma cells migration.	145 146
2	−	LA: Lupus La protein. SLE.	147
16	−	LRP1B: Low-density lipoprotein receptor-related protein 1B precursor 4599.	148
6	−	MAG: Myelin-associated glycoprotein precursor. MS.	149
4	−	MOG: Myelin-oligodendrocyte glycoprotein precursor. MS.	150
13	19	MYRF: Myelin regulatory factor. MS.	151
17	12	MYT1L: Myelin transcription factor 1-like protein. MS.	152
45	47	NBEL2: Neurobeachin-like protein 2 Role in neutrophil and NK cell function and pathogen defense.	153
27	−	NF1: Neurofibromin. neurofibromatosis.	154
44	47	NMDE4, Glutamate receptor ionotropic, NMDA 2D. Epileptic encephalopathy.	155
97	113	Obscurin: Heart disease.	156
26	39	SMCA4: Transcription activator BRG1. Esophageal, medulloblastoma, lung cancers.	157
62	113	SRRM2: Serine/arginine repetitive matrix protein 2. Thyroid carcinoma; Parkinson's disease.	158 159
15	−	STA13: StAR-related lipid transfer protein 13. Deleted in liver cancer 2 protein.	160
8	9	TGFB1: Transforming growth factor β-1 proprotein. Lupus nephritis in SLE Patients.	161
250	341	TITIN: Titin. Myocarditis, acute myocardial ischemia, cardiac arrest.	162
32	34	TRNK1: TPR and ankyrin repeat-containing protein 1. SLE. Neural development and differentiation.	163
12	−	TSP1: Thrombospondin-1. Inhibits tumor angiogenesis and suppresses tumor growth.	164
24	−	ZAN: Zonadhesin . Crucial role in sperm-zona adhesion. Sterility.	165
40	−	ZEP1: Zinc finger protein 40. Tum or-suppressive effects in prostate and nonsmall cell lung cancer.	166 167

Abbreviations: EBV, Epstein-Barr virus; DNA, deoxyribonucleic acid; MG, myasthenia gravis; MS, multiple sclerosis; SLE, systemic lupus erythematosus.

a Column A: number of shared peptides.

b Column B: number of shared peptides including multiple occurrences.

c Human proteins reported by UniProt entry names. Protein details, sequence, and aa length available at www.uniprot.org .

d Further references on the function/associated disease are available at UniProt, OMIM, and PubMed resources.

Also, the intense peptide sharing between immunoreactive EBV epitopes and KMT2C is of relevance. KMT2C not only may act as a tumor suppressor in leukemias and T-cell lymphomas, 75 76 but it is also implicated in bladder, breast, colorectal, endometrial, gastric, head and neck, lung, and liver cancer, and in medulloblastoma. 108

**Fig. 1:**
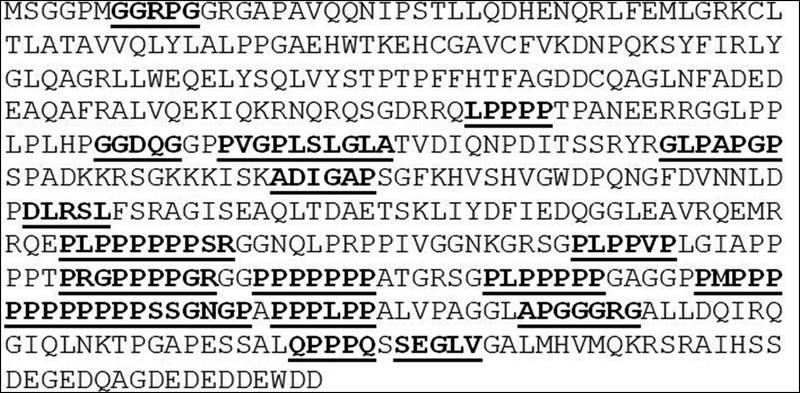
Distribution of EBV epitope-derived peptides throughout WASP primary aa sequence. WASP sequence from Uniprot ( http://www.uniprot.org/) . 51 EBV epitope-derived peptides are underlined and bold marked. EBV, Epstein-Barr virus; WASP, Wiskott-Aldrich syndrome protein.

Then, in spite of the lack of space, it is mandatory noting the harmful cross-reactivity platform represented by the peptide commonality between the immunoreactive EBV epitopes and Wiskott-Aldrich syndrome protein (WASP) ( Table 4 ). The 29 pentapeptides shared with EBV epitopes mainly occur throughout the central and COOH regulatory domains of the WASP primary sequence ( Fig. 1 , shared peptides in underlined bold character) and produce a “bull” for the EBV-activated immune system that is practically impossible not to hit. Hitting WASP can lead to lymphomagenesis. Indeed, WASP is a tumor suppressor frequently low or absent in anaplastic large cell lymphoma. 92 WASP deficiency relates to Wiskott-Aldrich syndrome (WAS). 109 110 111 112 WAS is characterized by eczema, thrombocytopenia, recurrent infections, immunodeficiency, neutropenia, and bloody diarrhea. 113 A large proportion of WAS patients develop autoimmunity and allergy since WASP appears to play an important role in the activation of CD4(+)CD25(+)FOXP3(+) natural regulatory T cells. 114 Even in the absence of typical clinical manifestations of WAS, a low expression of WASP associates with the pathogenesis of a subtype of inflammatory bowel disease. 115 Furthermore, deficiency of WASP associates with exacerbated experimental arthritis. 116

Overall, the peptide sharing between the immunoreactive EBV epitopes and KMT2D, KMT2C, and WASP proteins suffices to define the constellation of human diseases associated with EBV infection.

### The Pathological Implications of the Peptide Sharing between EBV Epitopes and Human Proteins: Various Cancers and Diseases

Table 4 illustrates that the EBV epitope-derived pentapeptides are widespread among the most disparate human proteins able to cause, when altered, a vast spectrum of diseases, from diabetes and sterility to myocarditis and death, 117 118 119 120 121 122 123 124 125 126 127 128 129 130 131 132 133 134 135 136 137 138 139 140 141 142 143 144 145 146 147 148 149 150 151 152 153 154 155 156 157 158 159 160 161 162 163 164 165 166 167 the latter two being possibly associated with the Titin imposing peptide sharing (250 shared pentapeptides).

## Discussion

We summarize here the vast peptide platform that, with impressive mathematical unexpectedness, connects immunoreactive EBV epitopes and human proteins.

Quantitatively, Table 1 shows that the peptide sharing does not obey to any theoretical probability expectations or constraints such as, for example, protein dimension. The case is best illustrated by the far upstream element-binding protein 2 (FUBP2; 711 aa) and the low-density lipoprotein receptor-related protein 1B (LRP1B; 4,599 aa). FUBP2 has 34 pentapeptides in common with the herpesviral proteome, whereas the much longer LRP1B shares 16 pentapeptides ( Table 4 ). That is, a high number of shared pentapeptides can be found in a protein irrespective of the protein length.

Pathologically, the peptide sharing between the immunoreactive EBV epitopes and the human proteome implies the possibility of cross-reactions and of a consequent wide spectrum of diseases, from lymphomas and leukemias to diabetes and spermatogenesis ( Tables 3 and 4 ). From this point of view, Tables 3 and 4 offer a scientific explanation of the clinical fact that EBV infection can trigger so many and so various diseases in so different and distant parts of the body. Moreover, given the number of human proteins involved in the sharing, the possibility of cross-reacting with a specific protein or group of proteins and inducing a specific disease or group of diseases will depend on the “when and where” the disease-associated protein(s) will be expressed. Consequently, the EBV diseasome will manifest with different diseases depending on the age of the subjects and on the immunological imprinting by previous pathogen infections, 168 thus explaining also why, once the immune system has been activated by EBV, some subjects will develop a lymphoma while other subjects contract diabetes or lupus or will die.

Immunologically, the vast peptide sharing between immunoreactive EBV epitopes and human proteins fails to support the theory of microbial or of human immunological specificity and nullifies the current concept of self-tolerance. Indeed, it was advanced in the “50s and still persists today the Burnet's hypothesis according to which self-tolerance is achieved by the so-called negative selection” of self-reactive lymphocytes. 169 170 171 That is, lymphocytes with specificity for peptide sequences that are expressed in the human host are hypothesized to be deleted from the immunological repertoire during fetal or early life to avoid self-reactivity and the consequent autoimmunity. Clearly, such a hypothesis breaks down in front of the pervasive peptide overlap between immunoreactive EBV epitopes and human proteins. If the “negative selection” assumptions were true, the self-reactive lymphocytes targeting the experimentally validated EBV epitopes described here and almost exclusively composed by peptides common to human proteins would have had to be eliminated from the immunological repertoire in the fetal life. It seems that the postulated deletion of potentially self-reactive lymphocytes did not occur. Similar results have been obtained analyzing hepatitis C virus and human papillomavirus immunoreactive epitopes. 172 173 Altogether, our data indicate that potentially self-reactive lymphocytes are regularly produced by the immune system. It seems that the immune system, under physiological conditions, does not engage reactions with self-proteins or pathogens just in virtue of their peptide commonality. As already discussed, 174 175 176 it seems that it is just the vast peptide commonality to confer or, better, to reify protein immunotolerance.

As a collateral note, we observe that, while Tables 1 and 2 militate against the assumption of a “negative selection” of self-reactive lymphocytes, Tables 3 and 4 also question the defensive role of the immune response. By definition, immune system attacks pathogenic enemies and protects self-entities. That is, it is assumed that the immune system is endowed with the capacity of discerning a pathogen antigen from a self-protein and of behaving consequentially by attacking the “foes” and defending the “friends.” Instead of being analyzed and defined as an aggregate of molecules organized into functional biological pathways, the immune system is considered as a “thinking entity” that sees, discriminates, decides, and then attacks. Against such an anthropomorphous view, the present mathematical and biochemical data document that pathogenic immune responses can routinely occur following infections, as already experimentally demonstrated. 177 178 Pathogenic autoantibodies—that are usually considered as rare phenomena due to the so-called “immunological holes” deriving from an incomplete negative selection of the self-reactive lymphocytes 169 170 171 or that, even, have been denied as pure fantasies 179 —seem to be the rule.

Tables 3 and 4 show that anti-EBV immunoreactivity can hit a myriad of human proteins that, when (epi)genetically altered, can lead to cancers, autoimmune diseases, and even death. Such cross-reactive potential explains why higher the anti-EBV IgG antibody titer, worse may be the disease prognosis and faster the disease progression as described by a continuum of reports since the 1970s. 16 17 18 19 20 21 22 23 24 25 26 27 28 29 30 31 32 33 34 That is, autoimmunity is not a matter of “rare immunological holes,” but it is intrinsic to the immune response that involves most of the human proteome by being most of the human proteome shared with microbial entities as a result of a long evolutionary path that from viruses and bacteria led to the eukaryotic cell. 180

In conclusion, this study highlights the necessity of reviewing the hypothesis of the “negative selection” of self-reactive lymphocytes and, at the same time, emphasizes the importance of the “peptide uniqueness” concept to develop immunotherapies against EBV infection, and infections in general. Only immunotherapies based on peptides uniquely owned by the infectious agents would offer high specificity as well as the advantage of a lack of adverse events in the human host. 39 181 182 183
