# Supplementary material for: From Anti-EBV Immune Responses to the EBV Diseasome via Cross-reactivity
Source: Glob Med Genet. 2020 Aug 31;7(2):51–63. doi: 10.1055/s-0040-1715641 (PMC7490125; doi:10.1055/s-0040-1715641)
Supplement: Supplementary file 1 — Supplementary Material [file 10-1055-s-0040-1715641-s2000010.pdf]

Supplementary Table S1 EBV immunome consisting of 3,197 immunopositive linear epitopes, assembled from IEDB [50], and listed by IEDB ID number.

130; 272; 351; 408; 950; 1090; 1219; 1284; 1296; 1432; 1433; 1434; 1511; 1518; 1612; 1716; 1997; 2390; 2742; 2743; 3004; 3005; 3537; 3538; 3579; 3586; 3600; 3628; 3713; 3732; 3772; 3782; 3785; 3892; 3951; 4289; 4344; 5002; 5043; 5303; 5316; 5317; 5326; 5439; 5677; 5678; 5679; 5713; 5718; 5899; 5915; 5938; 6136; 6482; 6564; 6568; 6611; 6808; 7353; 7358; 7751; 7835; 8120; 8191; 8378; 8395; 8396; 8397; 8905; 9644; 9646; 10105; 10448; 10561; 10830; 10856; 10858; 10888; 10926; 10929; 10963; 11388; 11421; 11422; 11460; 11525; 11651; 11804; 11805; 11806; 11807; 12183; 12325; 12419; 13483; 13628; 13701; 13748; 13859; 13893; 14052; 14635; 15051; 15141; 15168; 15827; 16044; 16548; 16876; 16878; 16978; 16979; 17110; 17111; 17600; 17619; 17620; 17635; 18031; 18110; 18328; 18357; 18409; 18433; 18438; 18446; 18462; 18587; 18588; 18650; 18884; 18946; 19672; 19674; 19704; 19737; 19738; 20011; 20020; 20023; 20487; 20788; 20892; 21252; 21697; 21719; 21723; 21771; 21839; 21870; 21968; 22159; 22162; 22447; 22976; 23103; 23324; 23325; 23434; 23449; 23550; 23994; 24097; 24170; 24525; 24533; 24535; 24536; 24666; 24667; 25098; 25192; 25237; 25351; 25756; 25942; 26408; 26480; 26538; 26761; 26897; 26981; 27103; 27118; 27161; 27301; 27375; 27423; 27611; 27955; 27992; 28135; 29332; 29466; 29467; 29468; 29469; 29472; 29618; 29720; 29721; 30164; 30266; 30430; 30431; 30951; 31071; 31155; 32853; 32945; 33207; 33208; 33209; 33483; 33650; 33865; 33866; 33867; 35158; 35162; 35210; 35533; 35694; 35887; 36605; 36656; 37000; 37153; 37308; 37499; 37533; 37769; 37887; 37938; 37949; 37959; 37960; 38052; 38427; 38458; 38460; 38514; 38633; 38647; 38669; 38670; 38851; 39030; 39079; 39080; 39102; 39335; 39432; 39634; 39681; 39790; 39890; 40474; 40572; 40573; 40687; 40710; 40845; 40972; 41113; 41147; 41508; 41628; 41841; 42110; 42525; 42941; 43109; 43444; 43517; 44181; 44333; 44860; 44862; 45378; 45379; 45380; 45466; 45499; 45501; 46498; 46673; 46895; 46923; 47613; 47636; 47760; 47807; 47838; 47846; 47957; 48320; 48472; 48486; 48735; 48736; 48737; 48738; 48752; 48836; 48852; 48864; 48876; 48901; 48946; 48948; 48974; 48975; 48976; 49007; 49008; 49056; 49190; 49257; 49258; 49442; 49539; 49593; 49594; 49752; 49864; 49956; 50128; 50129; 50130; 50223; 50250; 50298; 50329; 50502; 50669; 50940; 50944; 50989; 51037; 51177; 51178; 51245; 51425; 51534; 51558; 51685; 51794; 51795; 51946; 51947; 51969; 52078; 52142; 52268; 52367; 52582; 52792; 53127; 53128; 53129; 53148; 53195; 53359; 53881; 53886; 53963; 54330; 54331; 54367; 54548; 54728; 55150; 55151; 55251; 55252; 55253; 55254; 55295; 55298; 55299; 55317; 55326; 55327; 55336; 55363; 55529; 55585; 55592; 55610; 55619; 55620; 55657; 55670; 55684; 55718; 55734; 56144; 56390; 56433; 56506; 56511; 56523; 56563; 56650; 56651; 56897; 57170; 57560; 57755; 58070; 58192; 58193; 59084; 59132; 59432; 59551; 59875; 60219; 60264; 60373; 60782; 60791; 60930; 60931; 61040; 61477; 61577; 62305; 62445; 62446; 62508; 62509; 62621; 62738; 62739; 63155; 63546; 63843; 64763; 64794; 64942; 65466; 65746; 66322; 66323; 66324; 67007; 67130; 67131; 67299; 67349; 67350; 67416; 67419; 67456; 67484; 67505; 67506; 67891; 68229; 68422; 68517; 68518; 68561; 68579; 68853; 69309; 69558; 69559; 69731; 69835; 70014; 70017; 70251; 70315; 70413; 70466; 70529; 70624; 70901; 70932; 71181; 71968; 71997; 72028; 72029; 72030; 72213; 73221; 72240; 72251;72698; 72877; 72963; 72970; 73146; 73322; 73853; 73861; 73862; 74111; 74120; 74401; 74402; 74772; 74774; 74886; 74887; 75188; 75189; 75356; 75358; 75360; 75471; 75533; 75673; 75731; 76275; 76333; 77774; 77775; 78346; 78382; 78432; 78501; 78534; 79527; 79634; 80152; 80251; 80335; 80457; 80493; 82545; 82914; 82959; 84989; 85686; 86674; 86900; 86944; 87003; 87359; 87642; 88362; 89071; 89152; 89413; 92645; 93173; 93251; 93570; 93709; 94034; 94962; 95077; 95086; 95093; 95234; 95336; 95399; 95423; 95428; 95676; 97209; 97317; 97532; 97668; 97743; 98084; 98564; 98685; 98695; 98739; 98770; 98877; 98943; 99019; 99064; 99066; 101256; 101380; 101467; 101654; 101878; 102036; 102119; 102253; 105092; 105109; 105124; 105126; 105154; 105155; 105156; 105167; 105186; 105197; 105225; 105230; 105247; 105249; 105270; 105276; 105277; 105293; 105295; 105307; 105315; 105333; 105334; 105413; 105479; 105491; 105503; 105504; 105557; 105590; 105824; 106067; 106070; 106084; 107724; 107771; 107869; 108006; 108083; 108191; 108601; 108668; 108936; 113211; 114562; 114667; 115656; 118770; 118776; 118800; 118801; 118806; 118807; 118809; 118817; 118824; 118827; 118828; 118930; 118948; 118970; 118976; 118994; 119004; 119011; 119013; 119022; 119155; 119216; 119307; 119614; 119738; 120221; 120960; 124606; 124861; 125726; 125747; 125790; 126051; 126128; 126207; 126528; 126690; 126919; 126967; 126970; 126980; 126985; 126986; 126990; 126991; 127118; 127195; 127267; 127369; 127392; 127408; 127484; 127626; 127771; 127781; 127989; 127990; 128009; 132023; 132096; 132781; 132901; 134679; 136122; 136167; 136316; 136514; 136557; 136570; 136706; 137773; 138854; 138856; 138859; 138881; 138882; 138887; 138917; 138934; 141230; 141342; 144712; 144763; 144799; 149653; 149659; 149727; 149795; 149798; 149830; 149869; 149882; 149899; 150018; 150019; 158594; 164543; 167590; 181016; 181017; 182399; 182408; 186702; 186927; 187222; 187225; 191290; 193620; 193621; 193631; 193713; 193714; 193715; 193717; 193722; 193723; 193839; 193898; 193901; 193902; 193919; 193924; 193996; 194001; 194116; 194126; 194129; 194160; 194178; 194194; 194202; 194233; 194236; 194239; 194240; 194241; 194259; 194260; 194392; 194431; 194442; 194443; 194448; 194449; 194450; 194503; 194534; 194535; 194555; 194657; 227006; 227016; 227017; 227018; 227019; 227020; 227021; 227022; 227023; 227024; 227025; 227041; 227094; 227095; 227100; 227110; 227143; 227163; 227188; 227189; 227204; 227208; 227256; 227309; 227311; 227369; 227460; 227478; 227504; 227513; 227516; 227517; 227537; 227561; 227587; 227670; 227673; 227777; 227778; 229092; 229099; 229458; 230371; 230392; 230413; 230608; 230640; 230770; 230798; 230966; 231029; 231136; 231402; 231403; 231419; 231477; 231489; 231547; 231553; 231629; 231634; 231642; 231668; 231672; 231676; 231696; 231699; 231707; 231748; 231755; 231765; 231766; 231770; 231800; 231802; 231813; 231825; 231829; 231833; 231839; 231840; 231842; 231844; 231851; 231853; 231854; 231855; 231857; 231860; 231864; 231865; 231880; 231882; 231890; 231899; 231908; 231909; 231921; 231932; 231936; 231940; 231966; 231967; 231969; 231974; 231975; 231988; 231995; 231998; 232020; 232021; 232030; 232031; 232042; 232043; 232044; 232045; 232046; 232074; 232076; 232078; 232080; 232081; 232082; 232085; 232086; 232095; 232096; 232097; 232098; 232103; 232141; 232142; 232147; 232149; 232177; 232178; 232184; 232185; 232187; 232193; 232194; 232199; 232209; 232210; 232214; 232232; 232242; 232276; 232281; 232292; 232308; 232309; 232315; 232331; 232332; 232341; 232354; 232357; 232367; 232368; 232371; 232408; 232410; 232416; 232419; 232427; 232437; 232441; 232442; 232466; 232470; 232471; 232472; 232473; 232474; 233279; 237632; 237633; 237637; 237639; 237654; 237669; 237670; 237671; 237681; 237686; 237697; 237707; 237728; 237760; 237783; 237785; 237798; 237800; 237806; 237827; 237831; 237839; 237849; 237850; 237870; 237896; 237906; 237919; 237920; 237923; 237946; 237954; 237974; 237984; 237988; 238004; 238014; 238015; 238019; 238024; 238040; 244281; 419159; 419164; 419231; 419243; 419268; 419381; 419401; 419402; 419408; 429189; 431702; 432092; 436133; 503962; 503972; 503973; 503974; 503975; 503984; 503998; 504002; 504024; 504031; 504037; 535869; 540369; 540377; 540390; 540408; 540433; 540486; 540537; 540564; 540571; 540583; 540600; 540602; 540604; 540626; 540628; 540633; 548981; 548987; 548994; 550460; 595247; 595554; 607121; 653929; 657134; 672845; 675184; 676208; 678154; 678156; 682137; 682138; 682139; 682140; 686218; 688225; 693699; 693700; 693704; 693706; 693707; 693711; 693712; 693713; 693715; 693716; 693718; 693721; 693722; 693723; 693724; 693726; 693727; 693728; 693729; 693730; 693733; 693735; 693736; 693737; 693738; 693739; 693740; 693741; 693743; 693745; 693747; 693749; 693750; 693752; 693753; 693756; 693757; 693762; 693764; 693767; 693768; 693771; 693772; 693773; 693776; 693778; 693780; 693783; 693784; 693785; 693786; 693789; 693790; 693791; 693792; 693793; 693794; 693796; 693798; 693800; 693801; 693802; 693803; 693805; 693809; 693810; 693812; 693816; 693817; 693818; 693819; 693821; 693822; 693823; 693824; 693825; 693826; 693827; 693828; 693829; 693831; 693833; 693834; 693837; 693838; 693841; 693842; 693843; 693844; 693846; 693848; 693850; 693852; 693853; 693854; 693856; 693859; 693860; 693863; 693865; 693866; 693867; 693868; 693869; 693870; 693871; 693873; 693874; 693875; 693876; 693879; 693880; 693881; 693884; 693885; 693886; 693887; 693889; 693890; 693891; 693892; 693893; 693895; 693898; 693904; 693906; 693907; 693908; 693909; 693910; 693911; 693912; 693914; 693915; 693917; 693918; 693920; 693922; 693924; 693925; 693926; 693927; 693929; 693931; 693932; 693933; 693934; 693937; 693938; 693939; 693940; 693943; 693945; 693946; 693948; 693949; 693950; 693951; 693952; 693953; 693954; 693955; 693956; 693957; 693959; 693960; 693961; 693962; 693965; 693966; 693968; 693970; 693971; 693972; 693974; 693975; 693976; 693977; 693982; 693983; 693984; 693986; 693987; 693988; 693989; 693990; 693991; 693993; 693994; 693995; 693996; 693999; 694001; 694003; 694009; 694010; 694011; 694013; 694016; 694017; 694018; 694019; 694020; 694021; 694022; 694024; 694025; 694026; 694027; 694031; 694032; 694033; 694034; 694035; 694036; 694042; 694043; 694044; 694045; 694047; 694049; 694050; 694051; 694052; 694053; 694057; 694060; 694061; 694062; 694067; 694071; 694075; 694076; 694077; 694078; 694079; 694081; 694082; 694083; 694084; 694087; 694089; 694090; 694091; 694093; 694094; 694095; 694098; 694099; 694100; 694103; 694104; 694106; 694107; 694108; 694109; 694110; 694111; 694112; 694113; 694116; 694120; 694121; 694122; 694124; 694125; 694126; 694129; 694130; 694132; 694135; 694139; 694141; 694144; 694145; 694148; 694149; 694150; 694152; 694153; 694154; 694155; 694156; 694157; 694158; 694159; 694161; 694163; 694164; 694167; 694168; 694170; 694171; 694172; 694173; 694174; 694175; 694176; 694177; 694178; 694179; 694180; 694181; 694183; 694185; 694187; 694189; 694190; 694194; 694198; 694201; 694204; 694205; 694206; 694207; 694208; 694211; 694212; 694213; 694214; 694215; 694216; 694217;

694219; 694220; 694221; 694222; 694223; 694224; 694225; 694227; 694232; 694233; 694236; 694238; 694239; 694242; 694243; 694244; 694245; 694246; 694247; 694248; 694251; 694252; 694253; 694254; 694255; 694256; 694257; 694258; 694259; 694261; 694262; 694263; 694265; 694268; 694272; 694273; 694274; 694278; 694280; 694281; 694282; 694283; 694285; 694286; 694289; 694291; 694292; 694293; 694294; 694296; 694297; 694298; 694302; 694305; 694307; 694308; 694309; 694310; 694311; 694312; 694313; 694315; 694317; 694318; 694319; 694320; 694321; 694322; 694323; 694324; 694325; 694326; 694327; 694328; 694329; 694330; 694331; 694334; 694336; 694338; 694339; 694340; 694341; 694342; 694343; 694346; 694350; 694353; 694356; 694360; 694361; 694363; 694364; 694365; 694367; 694370; 694375; 694376; 694379; 694380; 694382; 694385; 694386; 694387; 694388; 694392; 694393; 694394; 694395; 694398; 694400; 694403; 694404; 694409; 694410; 694411; 694412; 694413; 694414; 694415; 694416; 694417; 694419; 694420; 694422; 694423; 694426; 694428; 694429; 694430; 694431; 694432; 694433; 694434; 694435; 694436; 694437; 694438; 694440; 694442; 694447; 694448; 694449; 694453; 694454; 694455; 694459; 694460; 694461; 694462; 694463; 694464; 694465; 694468; 694470; 694471; 694474; 694475; 694476; 694477; 694478; 694480; 694483; 694484; 694486; 694487; 694489; 694491; 694494; 694495; 694496; 694497; 694498; 694499; 694500; 694502; 694503; 694504; 694505; 694508; 694513; 694516; 694522; 694523; 694525; 694526; 694527; 694530; 694531; 694535; 694544; 694545; 694546; 694547; 694550; 694551; 694553; 694554; 694555; 694557; 694559; 694561; 694562; 694564; 694565; 694567; 694569; 694570; 694571; 694572; 694573; 694575; 694576; 694577; 694580; 694581; 694582; 694583; 694584; 694585; 694586; 694587; 694590; 694591; 694592; 694594; 694595; 694596; 694606; 694608; 694609; 694610; 694614; 694615; 694616; 694617; 694619; 694620; 694621; 694622; 694623; 694625; 694626; 694627; 694628; 694630; 694631; 694632; 694633; 694634; 694635; 694636; 694637; 694640; 694642; 694645; 694646; 694648; 694650; 694652; 694653; 694654; 694655; 694656; 694657; 694660; 694662; 694663; 694664; 694665; 694669; 694670; 694671; 694672; 694676; 694678; 694679; 694681; 694683; 694684; 694686; 694687; 694688; 694689; 694690; 694691; 694692; 694693; 694694; 694695; 694696; 694697; 694698; 694699; 694702; 694703; 694706; 694707; 694708; 694711; 694712; 694714; 694715; 694716; 694719; 694720; 694721; 694722; 694723; 694724; 694725; 694726; 694728; 694731; 694732; 694735; 694737; 694738; 694739; 694741; 694742; 694744; 694745; 694749; 694750; 694751; 694752; 694753; 694754; 694755; 694757; 694760; 694761; 694762; 694765; 694766; 694767; 694768; 694769; 694773; 694774; 694776; 694777; 694778; 694779; 694780; 694785; 694786; 694787; 694790; 694791; 694793; 694795; 694796; 694797; 694798; 694799; 694800; 694801; 694805; 694806; 694808; 694809; 694810; 694811; 694814; 694815; 694818; 694819; 694820; 694821; 694822; 694823; 694824; 694825; 694827; 694829; 694830; 694831; 694833; 694834; 694835; 694836; 694843; 694844; 694846; 694847; 694848; 694849; 694850; 694851; 694853; 694855; 694856; 694857; 694858; 694859; 694860; 694861; 694862; 694863; 694864; 694865; 694866; 694867; 694868; 694869; 694870; 694871; 694873; 694875; 694876; 694877; 694878; 694879; 694880; 694881; 694882; 694883; 694885; 694886; 694887; 694888; 694889; 694892; 694894; 694898; 694899; 694901; 694902; 694903; 694904; 694905; 694906; 694907; 694914; 694919; 694921; 694922; 694923; 694925; 694927; 694929; 694931; 694932; 694933; 694934; 694935; 694936; 694937; 694938; 694940; 694945; 694949; 694950; 694951; 694952; 694953; 694954; 694957; 694959; 694960; 694961; 694962; 694964; 694965; 694966; 694970; 694972; 694973; 694974; 694977; 694978; 694979; 694980; 694982; 694986; 694987; 694989; 694990; 694991; 694992; 694994; 694995; 694998; 695008; 695009; 695010; 695011; 695013; 695014; 695019; 695021; 695023; 695025; 695026; 695027; 695029; 695030; 695032; 695037; 695038; 695039; 695042; 695043; 695044; 695045; 695049; 695050; 695051; 695053; 695055; 695056; 695057; 695061; 695062; 695063; 695069; 695071; 695079; 695082; 695083; 695085; 695089; 695091; 695092; 695093; 695094; 695095; 695096; 695097; 695099; 695100; 695101; 695102; 695103; 695104; 695105; 695106; 695112; 695113; 695122; 695125; 695127; 695128; 695131; 695132; 695134; 695135; 695138; 695139; 695140; 695141; 695142; 695147; 695152; 695153; 695154; 695155; 695160; 695162; 695163; 695164; 695165; 695166; 695167; 695168; 695169; 695170; 695171; 695172; 695173; 695176; 695178; 695179; 695182; 695183; 695184; 695190; 695191; 695193; 695194; 695196; 695198; 695199; 695200; 695202; 695204; 695209; 695210; 695213; 695218; 695219; 695220; 695221; 695224; 695225; 695226; 695227; 695229; 695230; 695237; 695238; 695239; 695240; 695242; 695244; 695245; 695246; 695247; 695248; 695249; 695252; 695253; 695254; 695255; 695256; 695261; 695263; 695264; 695268; 695269; 695275; 695276; 695278; 695279; 695280; 695281; 695282; 695286; 695287; 695290; 695292; 695293; 695294; 695296; 695297; 695298; 695300; 695304; 695305; 695306; 695311; 695312; 695314; 695315; 695317; 695319; 695320; 695321; 695322; 695323; 695324; 695326; 695328; 695329; 695334; 695337; 695338; 695339; 695341; 695342; 695343; 695348; 695349; 695350; 695352; 695353; 695354; 695356; 695357; 695360; 695361; 695362; 695363; 695364; 695367; 695368; 695369; 695370; 695371; 695372; 695373; 695374; 695375; 695376; 695377; 695378; 695381; 695382; 695383; 695384; 695385; 695387; 695388; 695389; 695390; 695391; 695392; 695393; 695395; 695396; 695397; 695398; 695399; 695400; 695401; 695402; 695404; 695406; 695407; 695408; 695409; 695410; 695411; 695413; 695414; 695416; 695418; 695420; 695422; 695423; 695424; 695425; 695426; 695427; 695429; 695431; 695433; 695435; 695436; 695437; 695438; 695439; 695441; 695442; 695445; 695448; 695449; 695450; 695452; 695455; 695457; 695459; 695463; 695465; 695466; 695467; 695468; 695469; 695470; 695472; 695473; 695474; 695475; 695476; 695478; 695479; 695481; 695484; 695485; 695487; 695489; 695490; 695491; 695492; 695495; 695496; 695498; 695500; 695501; 695505; 695507; 695508; 695509; 695510; 695511; 695519; 695520; 695521; 695522; 695524; 695525; 695526; 695527; 695530; 695531; 695532; 695533; 695534; 695535; 695536; 695537; 695538; 695539; 695540; 695541; 695542; 695545; 695546; 695548; 695552; 695554; 695555; 695556; 695557; 695558; 695560; 695562; 695566; 695567; 695568; 695569; 695570; 695573; 695574; 695575; 695576; 695579; 695580; 695581; 695582; 695585; 695587; 695588; 695589; 695590; 695591; 695592; 695593; 695594; 695599; 695600; 695601; 695607; 695609; 695612; 695613; 695614; 695616; 695617; 695620; 695621; 695622; 695623; 695626; 695627; 695630; 695632; 695633; 695635; 695637; 695638; 695639; 695640; 695641; 695643; 695644; 695645; 695647; 695648; 695651; 695654; 695655; 695656; 695657; 695658; 695659; 695662; 695663; 695664; 695665; 695666; 695667; 695668; 695670; 695671; 695672; 695673; 695675; 695677; 695678; 695679; 695680; 695681; 695682; 695683; 695684; 695686; 695693; 695695; 695696; 695697; 695698; 695699; 695700; 695701; 695702; 695703; 695704; 695705; 695706; 695707; 695708; 695710; 695711; 695712; 695715; 695716; 695717; 695718; 695719; 695725; 695726; 695727; 695728; 695729; 695730; 695733; 695734; 695737; 695738; 695739; 695740; 695741; 695742; 695744; 695745; 695746; 695747; 695748; 695749; 695750; 695751; 695752; 695753; 695754; 695755; 695756; 695758; 695759; 695760; 695762; 695763; 695765; 695766; 695767; 695769; 695770; 695771; 695772; 695773; 695774; 695775; 695776; 695777; 695778; 695780; 695781; 695783; 695788; 695790; 695791; 695792; 695793; 695794; 695795; 695798; 695801; 695802; 695805; 695806; 695807; 695809; 695811; 695812; 695813; 695815; 695816; 695818; 695819; 695820; 695821; 695822; 695823; 695824; 695825; 695826; 695827; 695829; 695830; 695831; 695832; 695835; 695836; 695838; 695839; 695840; 695842; 695843; 695844; 695847; 695848; 695849; 695850; 695852; 695856; 695859; 695860; 695861; 695862; 695863; 695865; 695866; 695872; 695873; 695875; 695876; 695877; 695878; 695880; 695881; 695884; 695889; 695894; 695895; 695896; 695898; 695900; 695901; 695902; 695903; 695904; 695906; 695907; 695909; 695910; 695911; 695912; 695913; 695914; 695915; 695916; 695917; 695919; 695920; 695921; 695922; 695923; 695924; 695925; 695926; 695929; 695931; 695932; 695934; 695935; 695936; 695937; 695938; 695939; 695940; 695941; 695942; 695944; 695945; 695947; 695948; 695949; 695950; 695955; 695956; 695957; 695958; 695959; 695960; 695961; 695962; 695963; 695964; 695965; 695966; 695968; 695972; 695973; 695975; 695976; 695977; 695978; 695979; 695980; 695981; 695983; 695984; 695985; 695986; 695987; 695990; 695991; 695992; 695995; 695998; 695999; 696000; 696003; 696004; 696006; 696009; 696010; 696012; 696013; 696017; 696018; 696019; 696020; 696021; 696028; 696029; 696031; 696032; 696034; 696035; 696036; 696037; 696038; 696039; 696040; 696041; 696042; 696044; 696045; 696046; 696048; 696049; 696050; 696051; 696055; 696056; 696057; 696058; 696060; 696061; 696062; 696064; 696065; 696066; 696067; 696069; 696070; 696071; 696072; 696074; 696075; 696076; 696080; 696081; 696083; 696084; 696085; 696090; 696091; 696093; 696095; 696096; 696098; 696102; 696105; 696106; 696109; 696110; 696111; 696112; 696113; 696116; 696117; 696119; 696121; 696122; 696123; 696124; 696126; 696127; 696128; 696129; 696130; 696131; 696132; 696133; 696134; 696135; 696136; 696137; 696138; 696139; 696140; 696145; 696147; 696150; 696151; 696154; 696158; 696159; 696160; 696161; 696163; 696165; 696166; 696168; 696169; 696174; 696175; 696177; 696178; 696179; 696180; 696181; 696182; 696183; 696186; 696187; 696188; 696189; 696192; 696193; 696194; 696195; 696197; 696198; 696199; 696200; 696206; 696211; 696213; 696214; 696215; 696217; 696219; 696221; 696225; 696226; 696227; 696228; 696229; 696230; 696231; 696232; 696233; 696234; 696235; 696236; 696237; 696238; 696239; 696240; 696241; 696242; 696245; 696246; 696248; 696249; 696250; 696251; 696252; 696253; 696254; 696255; 696256; 696257; 696259; 696260; 696262; 696263; 696265; 696268; 696269; 696271; 696272; 696273; 696276; 696277; 696278; 696279; 696280; 696282; 696283; 696284; 696285; 696287; 696288; 696289; 696291; 696293; 696294; 696296; 696298; 696299; 696300; 696301; 696302; 696303; 696304; 696305; 696306; 696308; 696309; 696310; 696311; 696313; 696314; 696318; 696319; 696320; 696321; 696322; 696324; 696327; 696329; 696333; 696334; 696337; 696338; 696342; 696343; 696344; 696347; 696348; 696351; 696354; 696355; 696356; 696357; 696358; 696359; 696360; 696361; 696363; 696364; 696367; 696368; 696369; 696371; 696372; 696373; 696374; 696376; 696377; 696378; 696381; 696382; 696385; 696386; 696389; 696392; 696393; 696394; 696395; 696397; 696398; 696400; 696401;

696402; 696404; 696405; 696406; 696407; 696409; 696410; 696412; 696413; 696414; 696416; 696417; 696418; 696419; 696420; 696424; 696425; 696426; 696427; 696430; 696431; 696432; 696434; 696435; 696439; 696440; 696446; 696447; 696450; 696453; 696454; 696456; 696457; 696459; 696462; 696463; 696465; 696466; 696467; 696468; 696472; 696473; 696479; 696483; 696484; 696486; 696488; 696490; 696492; 696493; 696494; 696496; 696497; 696498; 696499; 696500; 696503; 696507; 696509; 696514; 696517; 696521; 696522; 696525; 696526; 696529; 696531; 696532; 696533; 696534; 696536; 696538; 696539; 696540; 696542; 696543; 696544; 696545; 696546; 696547; 696548; 696549; 696552; 696553; 696555; 696556; 696557; 696559; 696560; 696561; 696563; 696564; 696565; 696566; 696567; 696568; 696569; 696570; 696571; 696574; 696575; 696576; 696579; 696581; 696582; 696583; 696585; 696586; 696587; 696588; 696589; 696592; 696595; 696599; 696600; 696602; 696605; 696606; 696608; 696609; 696611; 696613; 696615; 696616; 696619; 696620; 696621; 696622; 696623; 696624; 696626; 696627; 696629; 696630; 696633; 696634; 696635; 696636; 696639; 696640; 696644; 696650; 696651; 696652; 696653; 696654; 696655; 696656; 696658; 696660; 696664; 696665; 696666; 696670; 696672; 696674; 696675; 696677; 696678; 696681; 696682; 696683; 696684; 696685; 696687; 696689; 696697; 696700; 696701; 696702; 696703; 696710; 696711; 696716; 696718; 696719; 696722; 696723; 696729; 696730; 696731; 696733; 696734; 696735; 696736; 696737; 696739; 696740; 696745; 696746; 696747; 696748; 696749; 696750; 696751; 696753; 696754; 696755; 696756; 696757; 696758; 696759; 696760; 696762; 696763; 696764; 696766; 696767; 696771; 696773; 696774; 696776; 696777; 696778; 696780; 696783; 696784; 696785; 696787; 696788; 696789; 696791; 696794; 696795; 696796; 696797; 696798; 696799; 696800; 696801; 696802; 696803; 696804; 696806; 696807; 696808; 696812; 696813; 696818; 696819; 696822; 696827; 696828; 696829; 696830; 696831; 696832; 696833; 696835; 696839; 696844; 696845; 696846; 696848; 696849; 696850; 696851; 696852; 696853; 696854; 696856; 696857; 696859; 696861; 696862; 696863; 696864; 696865; 696866; 696867; 696871; 696874; 696875; 696876; 696877; 696880; 696881; 696883; 696886; 696888; 696889; 696890; 696891; 696892; 696895; 696896; 696898; 696900; 696901; 696902; 696903; 696904; 696908; 696909; 696914; 696916; 696917; 696918; 696919; 696923; 696924; 696927; 696928; 696929; 696930; 696931; 696932; 696934; 696935; 696936; 696937; 696943; 696944; 696945; 696946; 696948; 696949; 696951; 696952; 696953; 696954; 696957; 696958; 696959; 696961; 696962; 696963; 696970; 696971; 696972; 696973; 696974; 696975; 696976; 696977; 696978; 696979; 696980; 696983; 696984; 696985; 696986; 696997; 697000; 697001; 697002; 697003; 697004; 697006; 697007; 697008; 697009; 697012; 697013; 697015; 697016; 697017; 697018; 697019; 697020; 697021; 697022; 697023; 697024; 697026; 697027; 697029; 697030; 697032; 697033; 697036; 697039; 697040; 697042; 697043; 697044; 697045; 697046; 697050; 697051; 697052; 697053; 697054; 697055; 697056; 697057; 697058; 697059; 697061; 697063; 697065; 697066; 745459; 745467; 745468; 745478; 745479; 745480; 745481; 745491; 745503; 745504; 745507; 747194; 749416; 750156; 750177; 752950; 753257; 753261; 753262; 753269; 753272; 769602; 769737; 769739; 858179; 858188; 858269; 889912; 890842; 892246; 892317; 892893; 892951; 896128; 899328; 899655; 901245; 901682; 901996; 903350; 903869; 904179; 904373; 904706; 905277; 905725; 905858; 906292; 907160; 907462; 908430; 908489; 910573; 934271; 936806

Abbreviations: EBV, Epstein-Barr virus; IEDB, immune epitope DataBase.
Source: Epitope experimental details and references are available at www.iedb.org.
